# Supplementary figures and images for: The impact of individual perceptual and cognitive factors on collective states in a data-driven fish school model
Source: PLoS Comput Biol. 2022 Mar 2;18(3):e1009437. doi: 10.1371/journal.pcbi.1009437 (PMC8932591; doi:10.1371/journal.pcbi.1009437)

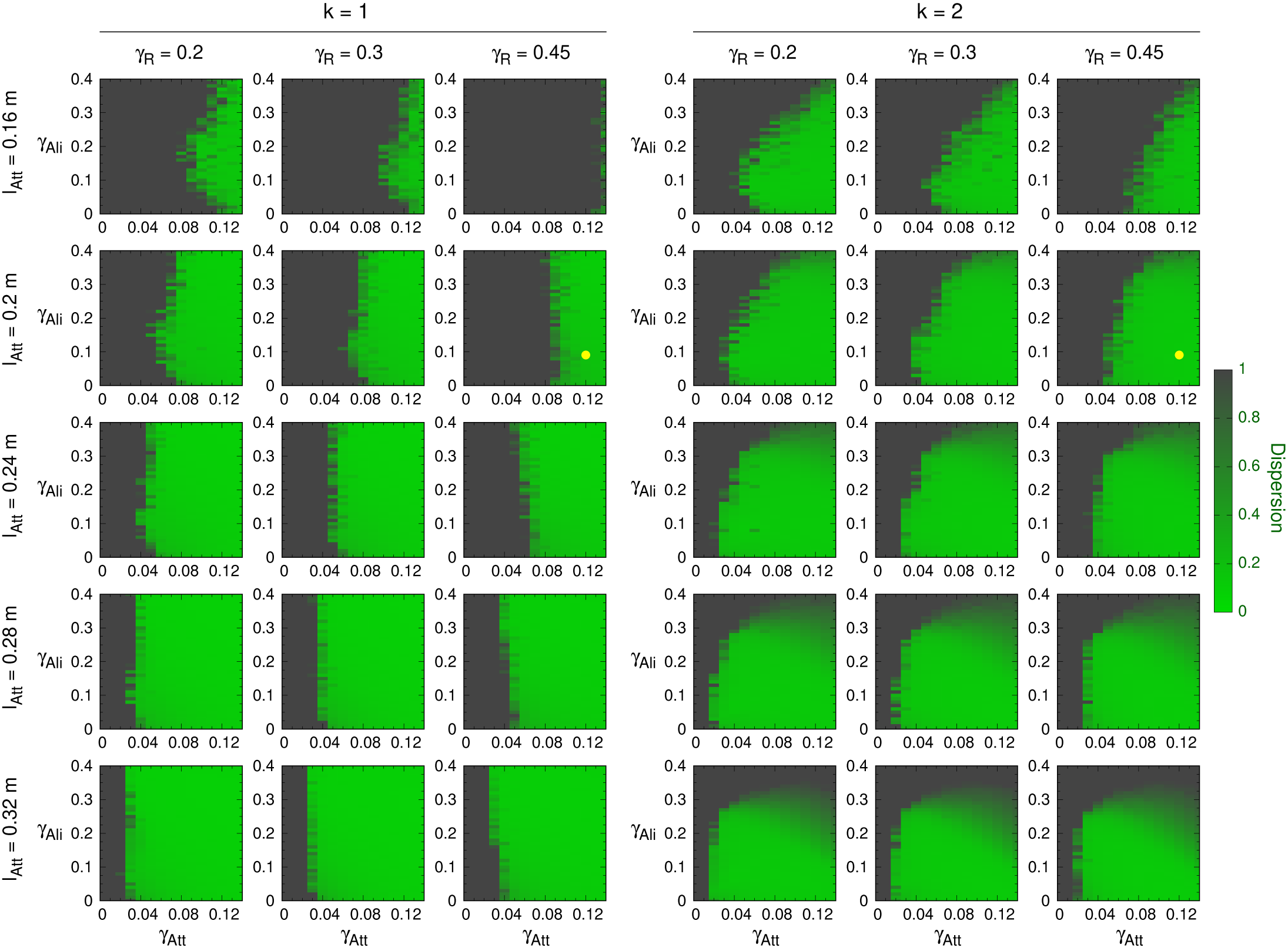

Supplement: S1 Fig — Each row corresponds to a given value of the interaction range; from top to bottom: lAtt = lAli = 0.16, 0.2, 0.24, 0.28, and 0.32 m. Each column corresponds to a given value of random fluctuations; from left to right: γR = 0.2, 0.3, and 0.45. Gray color corresponds to high dispersion (D > 1 m), green color to swarming phase. Intermediate color from gray to green correspond to the transition layer from dispersion to swarming. Values extracted from experiments with pairs of H. rhodostomus swimming in a small circular tank are lAtt = lAli = 0.2 m and γR = 0.45, denoted by a yellow dot in second row, columns 1 (k = 1) and 4 (k = 2). Figures appear pixelized because simulations are extremely long. We used a fine discretization with Δγ = 10−2 in both axes. (TIF) [file pcbi.1009437.s008.tif]

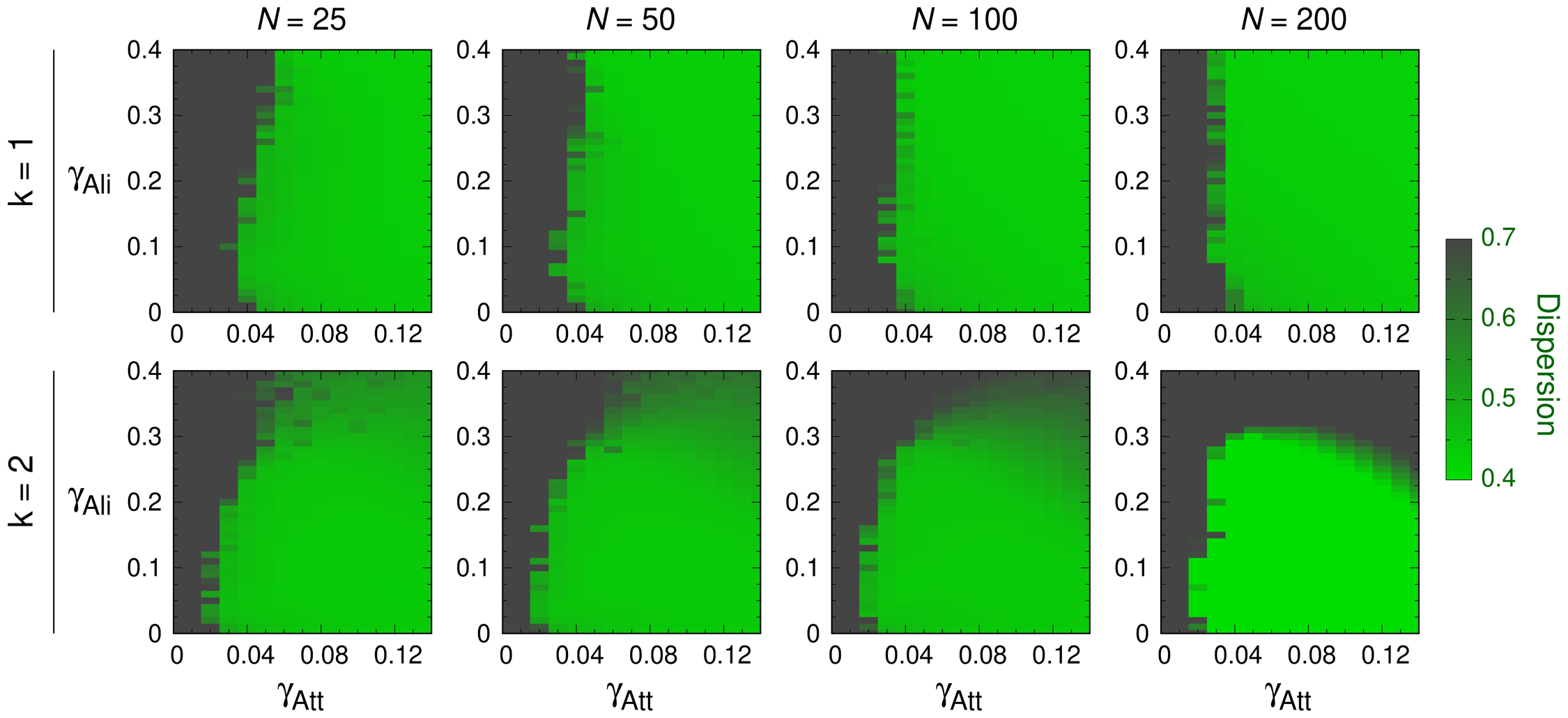

Supplement: S2 Fig — Gray color corresponds to region of high dispersion (D > 0.7 m), green color to swarming phase. Intermediate color from gray to green correspond to the transition layer from dispersion to swarming. Group sizes are, from left to right, N = 25, 50, 100, and 200 fish. First row: k = 1, second row: k = 2. We used lAtt = lAli = 0.28 m and γR = 0.2, as for Figs 3 to 6. (TIF) [file pcbi.1009437.s009.tif]

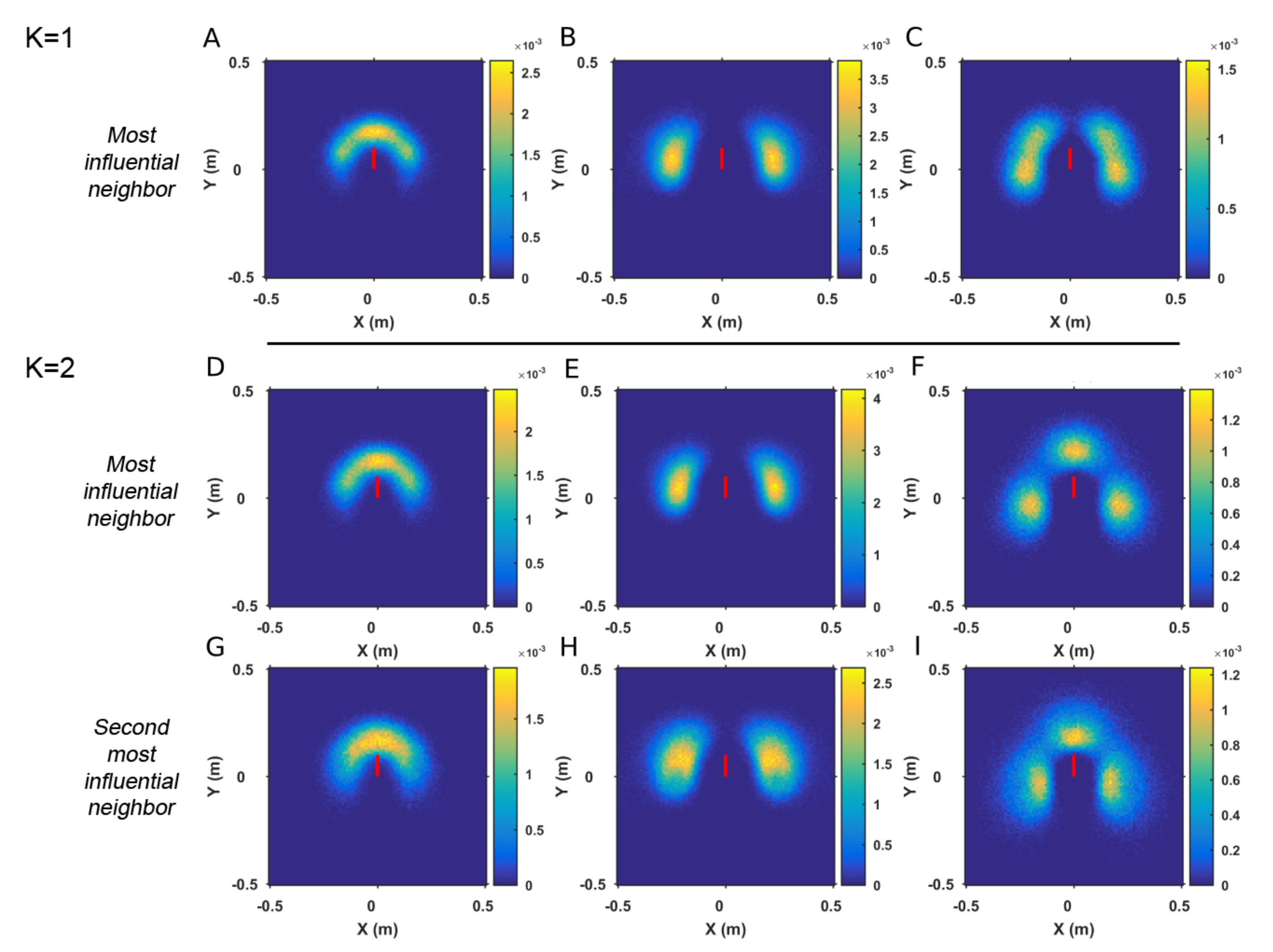

Supplement: S3 Fig — (ABC) Density maps of the relative position of the most influential neighbor of a fish when k = 1 in (A) the schooling state (γAtt = 0.03, γAli = 0.2), (B) the milling state (γAtt = 0.04, γAli = 0.04), and (C) the swarming state (γAtt = 0.1, γAli = 0.05). (D–I) Density maps of the relative position of (DEF) the most influential neighbor and (GHI) the second most influential neighbor of a fish, when k = 2, in (DG) the schooling state (γAtt = 0.02, γAli = 0.1), (EH) the milling state (γAtt = 0.02, γAli = 0.025), and (FI) the swarming state (γAtt = 0.1, γAli = 0.05). Each subgraph corresponds to the average of 2 runs, each run lasting about 1000 s (corresponding to 2000 kicks per fish), in which the first 200 s have been discarded. Bin size is 0.01 × 0.01 m2. The focal fish is represented by a short red line located at the origin (0, 0) and whose heading is pointing to the north. For all cases, N = 100, lAtt = lAli = 0.28 m, and γR = 0.2. (TIF) [file pcbi.1009437.s010.tif]

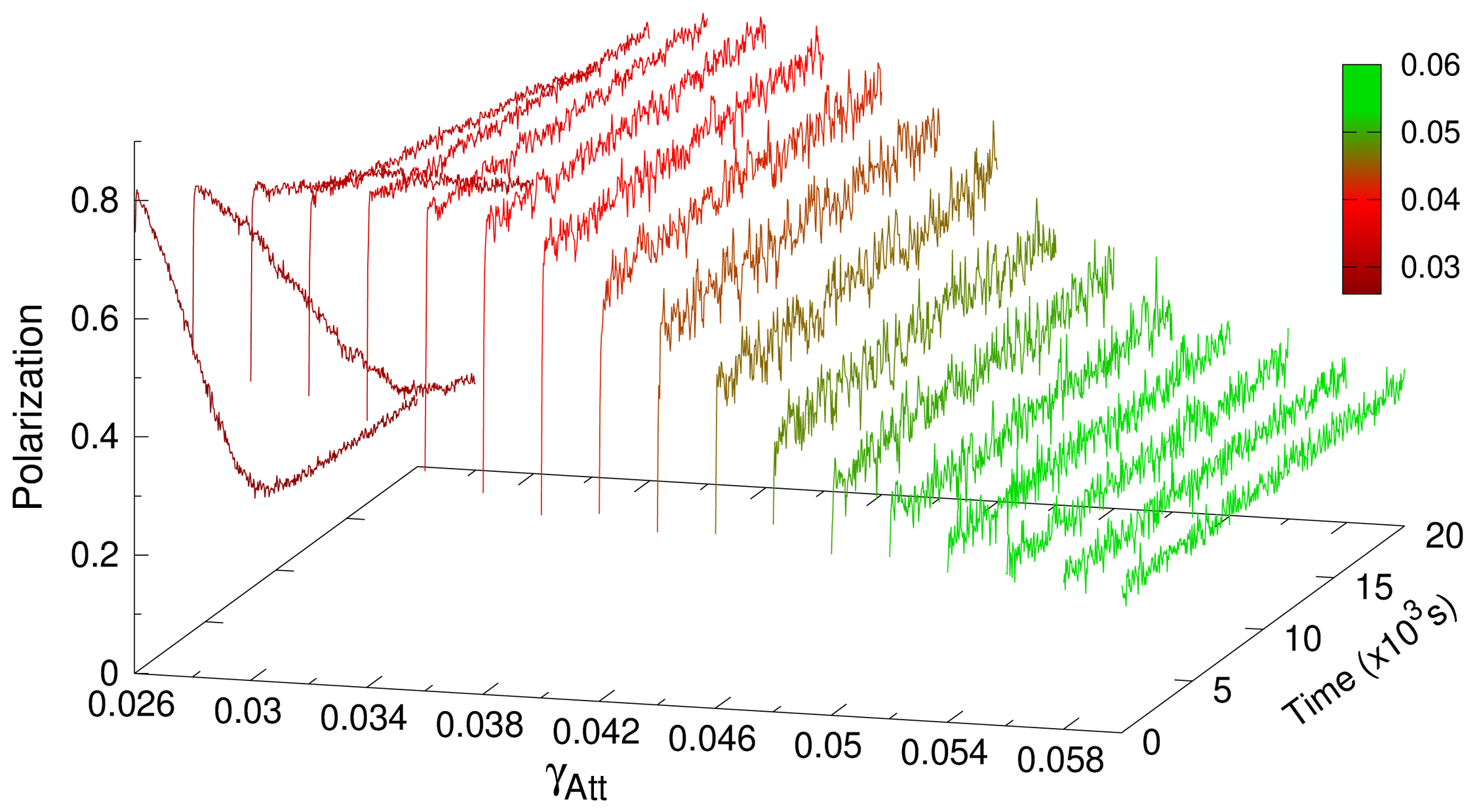

Supplement: S4 Fig — Each line is the mean polarization of the whole groups of N = 100 fish, averaged over 100 runs of 20.000 s of duration, for different values of γAtt ∈ [0.026, 0.06], i.e., across the schooling region shown in Fig 4, for γAli = 0.2. Line color denotes the phase region in which the point (γAtt, γAli) is located. Time instants are plotted only each 100 times. The value γAtt = 0.034 seems to be the threshold of the attraction strength below which the curve of the polarization decreases more or less rapidly to the value of no polarization (≈ 0.1), and above which the curve seems to remain constant in time, at a smaller height at larger values of γAtt. (TIF) [file pcbi.1009437.s011.tif]

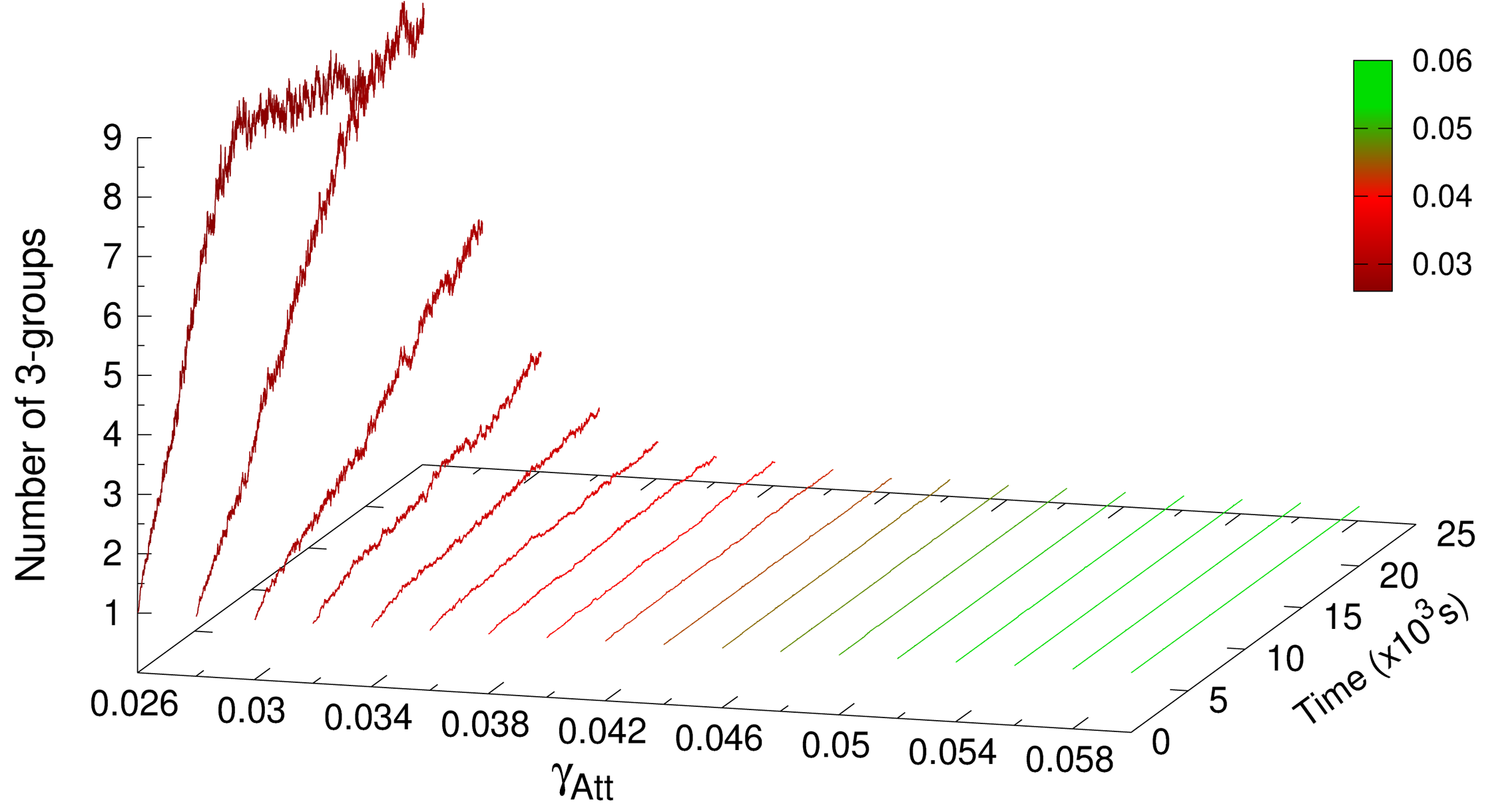

Supplement: S5 Fig — Each line is the mean number of 3-groups averaged over 100 runs of 20.000 s of duration, for different values of γAtt ∈ [0.026, 0.06], i.e., across the schooling region shown in Fig 4, for γAli = 0.2. Line color denotes the phase region in which the point (γAtt, γAli) is located. The partition of the N fish into 3-groups is done recursively: start with a fish i and put in its group its three nearest neighbors, then the three nearest neighbors of each nearest neighbor of i, and so on. When all the new neighbors of an iteration are already in the group, this 3-group is completed. Take another fish j which is not in a previous 3-group and repeat the process. Once all individuals belong to a 3-group, stop the process and count the number of 3-groups. (TIF) [file pcbi.1009437.s012.tif]

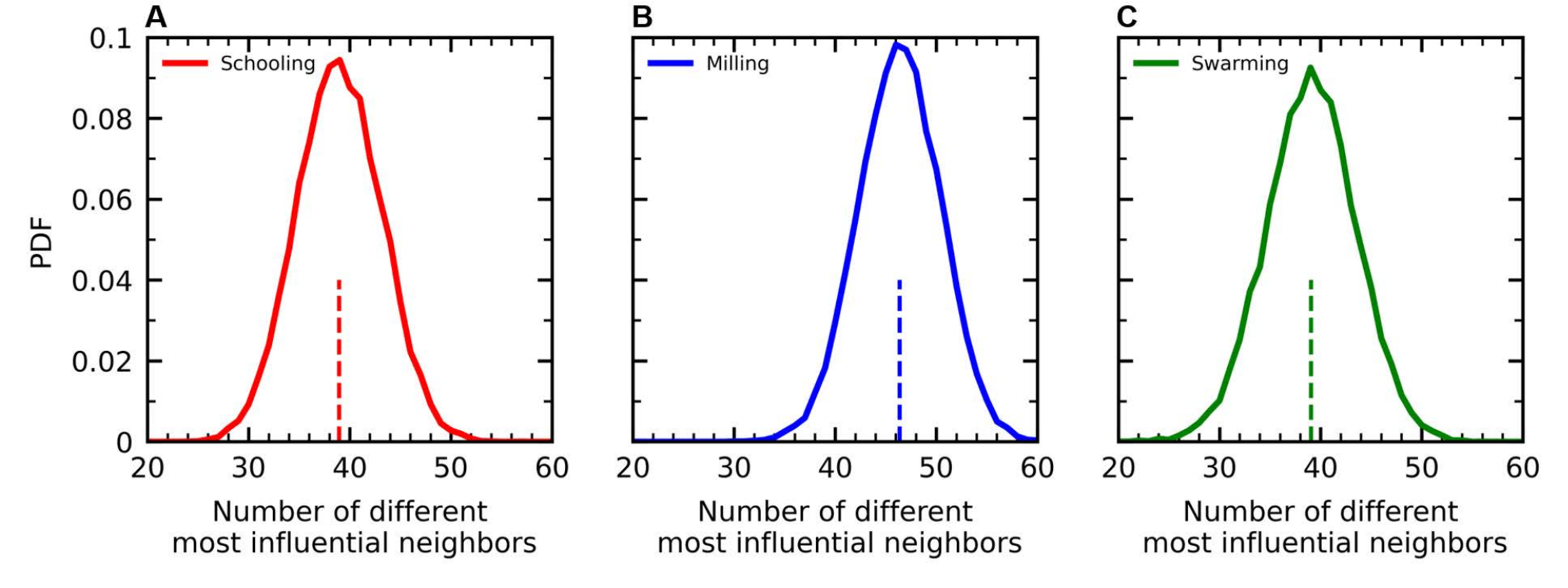

Supplement: S6 Fig — (A) Schooling state: γAtt = 0.035, γAli = 0.2; (B) Milling state: γAtt = 0.04, γAli = 0.05; (C) Swarming state: γAtt = 0.07, γAli = 0.1. PDFs and mean values are derived from 30 simulation runs, each of 1000 s duration, and sampled at each second in the time interval [200, 1000] (30 × 800 = 24000 data sampled per graph, leading to an uncertainty for the corresponding mean of order 0.1%). For all cases, the group size is N = 100, the intensity of random fluctuation is γR = 0.2, and the social interaction ranges are lAli = lAli = 0.28 m. We note that there are significantly more different most influential neighbors in the milling state (mean close to 46) than in the 2 other states (mean around 39). (TIF) [file pcbi.1009437.s013.tif]

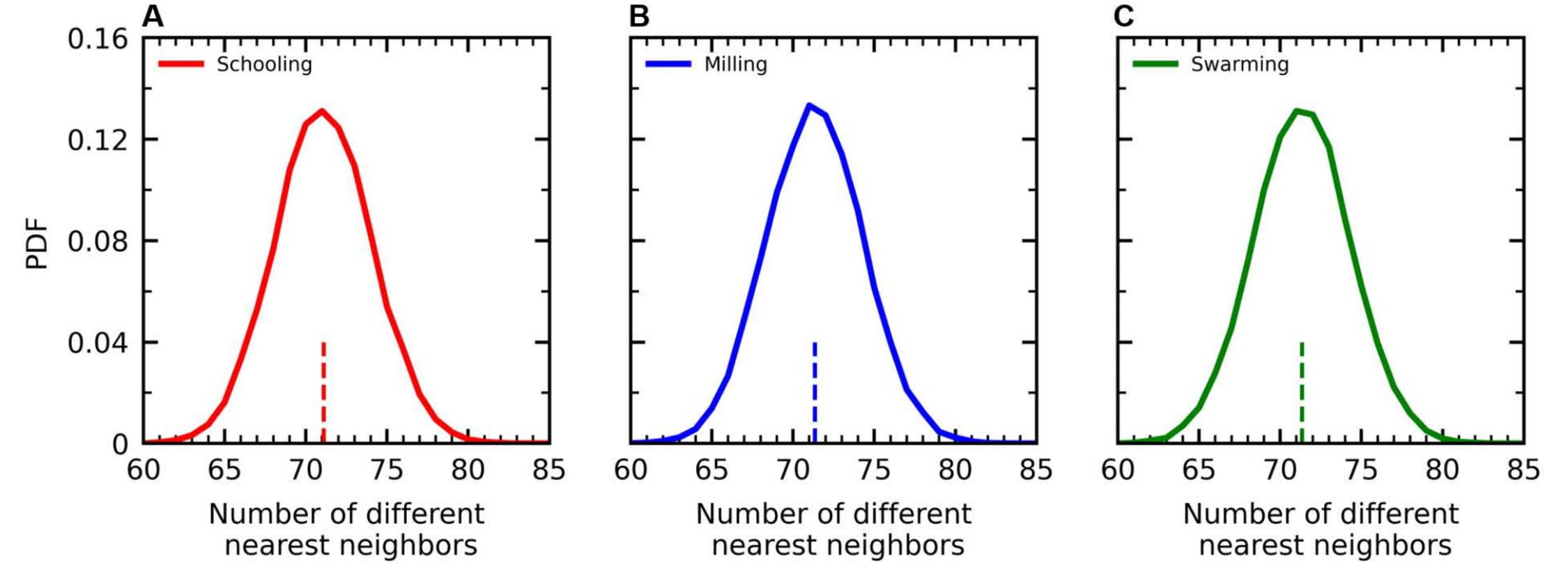

Supplement: S7 Fig — (A) Schooling state: γAtt = 0.035, γAli = 0.2; (B) Milling state: γAtt = 0.04, γAli = 0.05; (C) Swarming state: γAtt = 0.07, γAli = 0.1. PDFs and mean values are derived from 30 simulation runs, each of 1000 s duration, and sampled at each second in the time interval [200, 1000] (24000 data sampled per graph; uncertainty for the corresponding mean less than 0.1%). For all cases, the group size is N = 100, the intensity of random fluctuation is γR = 0.2, and the social interaction ranges are lAli = lAli = 0.28 m. We find that the mean number of different nearest neighbors is close to 71 in the three collective states, and is hence much larger than the corresponding number of different most influential neighbors. (TIF) [file pcbi.1009437.s014.tif]
