# Supplementary material for: The impact of individual perceptual and cognitive factors on collective states in a data-driven fish school model
Source: PLoS Comput Biol. 2022 Mar 2;18(3):e1009437. doi: 10.1371/journal.pcbi.1009437 (PMC8932591; doi:10.1371/journal.pcbi.1009437)
Supplement: Attachment — Submitted filename: review_Wang_etal.pdf [file pcbi.1009437.s015.pdf]

I have reviewed the paper "*The impact of individual perceptual and cognitive factors on collective states in a data-driven fish school model*" by W. Wang, R. Escobedo, S. Sanchez, C. Sire, Z. Han and G. Theraulaz.

The authors present a computational model inspired in the swimming behavior of fish (so-called "burst-and-coast"). This model considers social interactions of two types: attraction and alignment. The interaction neighborhood is defined by the k most influential neighbors (the authors study the case k=1 and k=2, as they explain that it is enough to observe/reproduce real fish behavior). The authors then quantify in a very systematic manner the collective behavior observed in the numerical simulations by computing three main collective observables: the group dispersion D(t), the group polarization P(t) and the milling index M(t). The authors identify five qualitatively different regions in the parameter space defined by the intensity of the social forces. These regions are: region I (schooling), region II (milling), region III (swarming), a region where the group is non-cohesive and a region where there is an intermittence between schooling and milling, called region I-II. The authors visualize their results by showing i) heat-maps of the parameter space (Figure 4), ii) time series of the observables (Figure 5) and detailed cuts of the heat-maps of the parameter space (Figure 6), something that helps the reader to understand clearly the observed collective patterns described in the results section. Finally, the authors study in a detailed way how the first results are modified when changing: a) the interaction ranges, b) the intensity of fluctuations and c) the system size.

The paper is well written, and all results are well explained. The conclusions seem reasonable within the context of the study and I think that it is suitable to be published in PLOS Computational Biology. I do, however, have a few comments and suggestions for the authors to consider:

1) The presented model has many parameters, something that allows the existence of multiple collective patterns by varying some of them, like the authors show in the paper. It would be good if the authors explain shortly in the discussion section if this model could be used to describe either other fish species, or even other animal species that perform intermittent motion, analogous to the "burst-and-coast" motion observed in fish.

2) Could the authors explain what would change if they consider that individuals move with variable speed, as suggested by P. P. Klamser et al (Frontiers in Physics, 9:715996, 2021). So far, the proposed model considers that the individuals move with constant speed between consecutive "kicks", as seen in equation (2).

3) In the results section, in line 273, the authors report that "The first remarkable result is that, despite the discrete character of the burst-and-coast swimming mode, the group of fish remains cohesive in a large region of the parameter space, even when fish interact only with their most influential neighbor, as testified by the wide regions of Figs. 3A and 3D." I wonder if this result is due to a few individuals playing the role of most influential neighbor to most of the members of the group, something that would explain the group staying together (and thus it would not be a surprise). This could be checked by the authors by computing the number of times that an individual plays the role of most influential neighbor to others (as a time series and/or in average).

Minor remark:
A) the arrow of angle \phi_i^n is inversed in Figure 1B.

Other than these points/doubts, I believe the paper contributes to the general understanding of self-organization dynamics in collective animal behavior. The data, approaches and model are well analyzed, developed, and presented. I thus support its publication in PLOS Computational Biology.
