# Supplementary material for: The impact of individual perceptual and cognitive factors on collective states in a data-driven fish school model
Source: PLoS Comput Biol. 2022 Mar 2;18(3):e1009437. doi: 10.1371/journal.pcbi.1009437 (PMC8932591; doi:10.1371/journal.pcbi.1009437)
Supplement: Attachment — Submitted filename: 0Rebuttal_Letter_Wang_et_al_R1.pdf [file pcbi.1009437.s016.pdf]

Dear Editor,

Please find attached the revised manuscript "The impact of individual perceptual and cognitive factors on collective states in a data-driven fish school model" by W. Wang, R. Escobedo, S. Sanchez, C. Sire, Z. Han, and G. Theraulaz.

We would like to thank you for the opportunity to revise our manuscript and the three reviewers for their constructive reviews and for the suggestions for improving our manuscript.

Please find below the detailed replies to the referees and the list of changes we have made to our manuscript.

Sincerely,

The authors

Reviewer's Responses to Questions

Reviewers have raised a total of 12 points that we reproduce here in black italicized text. Our answers are given in blue.

Reviewer #1:

I have reviewed the paper "The impact of individual perceptual and cognitive factors on collective states in a data-driven fish school model" by W. Wang, R. Escobedo, S. Sanchez, C. Sire, Z. Han and G. Theraulaz.

The authors present a computational model inspired in the swimming behavior of fish (so-called "burst-and-coast"). This model considers social interactions of two types: attraction and alignment. The interaction neighborhood is defined by the k most influential neighbors (the authors study the case k=1 and k=2, as they explain that it is enough to observe/reproduce real fish behavior). The authors then quantify in a very systematic manner the collective behavior observed in the numerical simulations by computing three main collective observables: the group dispersion D(t), the group polarization P(t) and the milling index M(t). The authors identify five qualitatively different regions in the parameter space defined by the intensity of the social forces. These regions are: region I (schooling), region II (milling), region III (swarming), a region where the group is non-cohesive and a region where there is an intermittence between schooling and milling, called region I-II. The authors visualize their results by showing i) heat-maps of the parameter space (Figure 4), ii) time series of the observables (Figure 5) and detailed cuts of the heat-maps of the parameter space (Figure 6), something that helps the reader to understand clearly the observed collective patterns described in the results section. Finally, the authors study in a detailed way how the first results are modified when changing: a) the interaction ranges, b) the intensity of fluctuations and c) the system size.

The paper is well written, and all results are well explained. The conclusions seem reasonable within the context of the study and I think that it is suitable to be published in PLOS Computational Biology.

A: We wish to thank the first referee for her/his positive review and for her/his helpful comments. We like that the referee considers that the article has been well written and the results well explained. We have indeed taken special care in writing and organizing the results to give them a biological meaning.

I do, however, have a few comments and suggestions for the authors to consider:

#1.1 *The presented model has many parameters, something that allows the existence of multiple collective patterns by varying some of them, like the authors show in the paper. It would be good if the authors explain shortly in the discussion section if this model could be used to describe either other fish species, or even other animal species that perform intermittent motion, analogous to the "burst-and-coast" motion observed in fish.*

A: The referee is right and her/his comment underlines the ability of the model to reproduce multiple collective patterns by simply adjusting the values of the parameters that govern the way individual fish interact with each other within a school. This is indeed a characteristic of the particular species of fish (*H. rhodostomus*) that has been modeled which accounts for the modulation of behavioral interactions between fish by environmental conditions such as variation of light that directly affect the perception of individuals. Our article precisely investigates how transitions between collective states take place, by, *e.g.*, reducing or increasing the respective intensities of attraction and alignment behavior between individuals. It is also quite natural to ask whether the results obtained for this species of fish can be generalized to other species.

However, the answer to this question strongly depends on the specific form of the social interaction functions. In a recent work, we applied the same procedure used to derive the *H. rhodostomus*' model to the zebrafish (*Danio rerio*), a species of fish that also performs burst-and-coast swimming (see Escobedo *et al.*, Phil. Trans. Roy. Soc. B, 2020, Ref. 44). The part of the model describing individual fish swimming is exactly the same in both species, as well as the way they combine the social interactions, showing that this part of the model can be applied to other fish species performing intermittent swimming. However, the shape of the social interaction functions reconstructed from the analyses of the trajectory data is completely different: interaction ranges in *H. rhodostomus* are much larger than in *D. rerio*, a feature that deeply affects the behavioral phases that emerge at the collective level.

In connection to the similar point #3.1 of reviewer #3, we have added two paragraphs addressing these points at the very beginning of section 3, which indeed provides clearer motivations for studying the phase diagram of the model (in addition to the obvious theoretical interest). In addition, we have added a short mention of these motivations in the discussion (around line 648).

#1.2. *Could the authors explain what would change if they consider that individuals move with variable speed, as suggested by P. P. Klamser et al (Frontiers in Physics, 9:715996, 2021). So far, the proposed model considers that the individuals move with constant speed between consecutive "kicks", as seen in equation (2).*

A: We thank the reviewer for pointing out the recently published paper by Klamser *et al*. (reference added), that we were not aware of. Indeed, speed variation is taken into account in the model, as shown precisely in Eq. (2), where the kick length and duration l_i and τ_i are (both) sampled from the bell-shaped distribution observed in the experiments, and therefore change, at each kick of each fish. Note also that the speed varies along each kick (*i.e.*, during the gliding phase) according to a decreasing exponential. Like the rest of the model, all these characteristics of the velocity are not arbitrary and have been extracted from the experimental data (see Calovi *et al.*, Plos Comput Biol, 2018, Ref. 43). We however agree with the referee that omitting these details do not help the reader to fully understand the model. We have thus added an explicit description in the text, in the paragraph following Equations (1)-(3):

"Kick lengths and durations l_i^n and τ_i^n are sampled from bell-shaped distributions obtained in the experiments, where the mean values l = 7cm and τ = 0.5s were found [43], as well as the speed maxima measured right after a kick, whose mean is v_0 = 14cm/s. During the gliding phase of the kick, fish speed decreases quasi-exponentially with a relaxation time measured to be $\tau_0 \approx$ 0.8 s, so that $v(t) = v_0 e^{-t/\tau_0}$ until the next kicking instant."

We have also modified the first paragraph of the section to mention explicitly that speed is not constant in our model as follows:

"Consecutive kicks of a single fish do not necessarily have the same length, duration and speed. When swimming in groups, kicks of different fish are asynchronous and of different length, duration and speed."

#1.3. *In the results section, in line 273, the authors report that "The first remarkable result is that, despite the discrete character of the burst-and-coast swimming mode, the group of fish remains cohesive in a large region of the parameter space, even when fish interact only with their most influential neighbor, as testified by the wide regions of Figs. 3A and 3D." I wonder if this result is due to a few individuals playing the role of most influential neighbor to most of the members of the group, something that would explain the group staying together (and thus it would not be a surprise). This could be checked by the authors by computing the number of times that an individual plays the role of most influential neighbor to others (as a time series and/or in average).*

A: The reviewer raises an excellent point which motivated us to compute the probability distribution function (PDF) of the number of different most influential neighbors (DMIN) at a given time, and in the three important collective phases (swarming; schooling; milling). These results are presented in the new Fig. S6. We find that the DMIN constitute roughly 39% (swarming & schooling; difference is not significant) or 46% (milling) of fish. Hence, the number of DMIN is certainly not (very) small (and should scale proportionally with N, anyway). Interestingly enough, the milling phase is characterized by more DMIN than the 2 other phases (which are indiscernible under this criterion), possibly because more information exchange within the school is needed to ensure the cohesiveness, alignment, *and* collective rotation of the school.

In the new Fig. S7, and again motivated by the reviewer's remark, we have also measured the PDF of the number of different first nearest neighbors (DFNN; for a physical attractive interaction only depending on distance, they would coincide with the DMIN). Interestingly, we find that there are much more DFNNs (71 on average, irrespectively of the collective phase) than DMINs. So, in a sense, the intuition of the referee is validated: the notion of DMIN allows to efficiently stabilize the school by having fish interacting with much less relevant neighbors at a given time than with the naïve notion of DFNNs.

Along figure S6 and S7, we have added a paragraph commenting these results just before the conclusion of section 3.1, around line 476 ("It is remarkable…") and also slightly extended this section conclusion. We also explicitly acknowledge the input from Reviewer #1 in the final Acknowledgments.

#1.4. *Minor remark: A) the arrow of angle \phi_i^n is inversed in Figure 1B.*

A: We thank the reviewer for this accurate remark! The figure has been corrected.

Other than these points/doubts, I believe the paper contributes to the general understanding of self-organization dynamics in collective animal behavior. The data, approaches and model are well analyzed, developed, and presented. I thus support its publication in PLOS Computational Biology.

A: We thank the reviewer for her/his support and again for her/his comments and suggestions that helped us to improve the manuscript.

Reviewer #2:

The authors study numerically the properties of a flocking model that they used previously to describe fish school data. The focus is on the impact of modeling elements that the authors interpret as regulating perceptual and cognitive factors. Special attention is paid to the number of influential neighbors, limiting this number to even two and one. Collective behavior is characterized using two observables, the polarization and the milling index, in small group size with up 200 individuals (small in comparison to studies that aimed at characterizing the large scale properties, and "universal classes", of flocking models).
I found the results of interest, providing insight on the collective dynamics of fish schools of medium, and the manuscript clearly written.

There is, however, one issue that the authors could address, which is at the moment missing in the manuscript.

A: Before we address the reviewer's comments point-by-point, we would like to thank her/his positive general evaluation of the manuscript. We also would like to point out that, although other similar studies on collective behavior make use of much larger sizes as, *e.g.*, we did in the study of the marine fish *Kuhlia mugil* in Calovi *et al. New Journal of Physics*, 2014 (groups of up to 4000 individuals, Ref.61), *H. rhodostomus* is a freshwater fish and is rarely found in groups of more than 100 individuals.

#2.1 *The model has attraction and repulsion and both interactions are formulated via complicated expressions that depend on relative distance, orientation, and position, involving a large number of parameters (even if here several of them are kept constant). Furthermore, the authors indicate in the conclusions "Transition between swarming, schooling, and milling can be induced by varying the interaction ranges". While I do not disagree with this observation, I think it would be important to mention that it has been shown that these collective behaviors can be also induced in simple models that use a fixed interaction range and only attraction (i.e. no alignment) by varying the angle of the field of view, see Barberis et al. Phys. Rev. Lett. 117, 248001 (2016), where the transitions to different collective behaviors can be understood as instabilities.*

A: We agree with the reviewer that much simpler models exist that can separately produce behavioral patterns that look similar to those observed in real fish schools. Our intention is however not just to "reproduce" collective motion patterns, but to analyze the phase diagram of a **model that has been accurately built and validated from experimental data of a specific species,** so that the model reflects as much as possible the main features of a real biological phenomenon. This includes, essentially, the burst-and-coast swimming mode, whose description requires a quite small number of parameters (the kick length l_i, the kick duration τ_i, and the speed relaxation time during kick τ_0), and the specific form of the social interaction functions, which is also characterized by really few parameters (the attraction and alignment interaction intensities $\gamma_{Att}$and γ_{Ali}, and their respective range l_{Att} and l_{Ali}). This is not the case for the models reported in the literature, as for instance the Barberis & Peruani's model suggested by the reviewer. This is a continuous mean

field model with interactions given by arbitrary general functions, and where essential individual characteristics such as the discrete swimming mode and the specific form of the interactions are not considered.

#2.2 *Moreover, I wonder whether it is really necessary to have both, attraction and repulsion, and complicated expressions for both. I am not asking the authors to answer to this question (which I image require a separate study), but to comment on potential alternatives to obtain different collective dynamics, which could be of relevance in the study of animal groups (including fish groups).*

A: As already mentioned, the model, in particular the interaction functions, is not build *ad hoc* to reproduce different collective motion patterns. The shape of the social interaction functions between fish have been specifically extracted from experimental data in Calovi *et al.*, Plos Comput Biol, 2018 (Ref.43) and, at some point, don't leave room for manipulation without the risk of losing fidelity to what is observed in the reality. Both attraction and alignment are at play in this species. In the hypothetical case where an *ad hoc* model would be able to reproduce the observed behavioral patterns without one of the two kinds of interactions, the model would be unrealistic because both interactions have been measured, and therefore both interaction functions must be used and combined adequately to reproduce the real fish behavior. Expressions may appear complicated, but mostly only in their analytical form, not in the number of parameters involved.

Of course, the referee is right in that it would be of great interest to identify in these expressions the essential features that could give rise to the observed collective dynamics, for instance, the parity of the angular functions, or the fast or long decay of the range of interaction. We have started to investigate these issues with a generalized model.

Reviewer #3:

This paper describes models for fish schooling that are inspired by a data-drive model based on the collective behaviour of H. rhodostomus. This work goes to great detail in describing how the different model parameters influence the formation of different collective states. Further, these results are not just presented as is but, rather, each one is accompanied by the relevant intuitive explanation. The discussion nicely wraps up the paper by connecting these findings to relevant biological questions concerning fish schooling. In general, I would support publication after some of my concerns, listed below, have been lifted.

A: We thank the reviewer for her/his positive comments on our manuscript.

#3.1 *It is not clear enough if variation in different model parameters is meant to describe different fish species or dynamic variations in the species of study itself.*

- For the first case (describe a variety of species), how generalizable is this model expected to be for other species? The model is quite unique in that the most influential fish are not the nearest ones (either metrically or topologically) but rather those that are most different from the focal fish. It might be the case that other fish species use very different modes of schooling which would make much of the results here insignificant. Do the authors expect this behavior to appear in other species? What is the evidence for this?

- In the second case (i.e., the model is meant to describe variations in the same species), is there any evidence that these fish vary their interaction parameters in different circumstances? Which parameters are more likely to be controlled by the fish and which by the environment? Are all

parameters studied in this work of equal importance? If these parameter changes are not experimentally approachable then why should they interest the reader?

A: The model that is studied in this paper is intended to describe the diversity and plasticity of collective behavior in the same species of fish (*Hemigrammus rhodostomus*). However, as we explained previously in our answer to referee 1 (see *#1.1),* the structure of the burst-and-coast model can be used to describe other species of fish performing intermittent swimming. This is the case of zebra fish (*Dano rerio*). However, the social interaction functions are different from those measured in *Hemigrammus rhodostomus*. In particular the range of interactions is much shorter, contributing to explain the fact that zebra fish display a weaker collective social behavior than Hemigrammus. We discussed these points in a previous paper (Escobedo *et al.*, Phil. Trans. Roy. Soc. B, 2020, Ref.44).

In addition, for a given species, fish can indeed experience different interaction parameters depending of their age or of their environment (turbidity of the water, light intensity…). For instance, it was shown in Gautrais et al. (Ref.30) that the intensity of the alignment interaction for *Kuhlia mugil* is essentially proportional to the mean velocity of the school. Moreover, a recent experimental work has shown that the strength of interaction between individual fish in *H. rhodostomus* may change as a consequence of variation of light intensity (McKee et al. Ref.48). The authors have shown in darkness and under low illuminance, fish moved with low polarization and a large mean nearest-neighbor distance that spanned many body lengths, thus confirming some predictions of our model. However, the authors did not use the complete set of metrics that we used ourselves to quantify the collective phases and worked on small group sizes. In the same article, the authors showed that it was possible to change the intensity of the attraction behavior by manipulating the lateral-line system through exposure to a solution of neomycin sulfate. We plan to use the same type of experimental methods to test some predictions of the model on larger group sizes (around fifty individuals). In particular the illuminance of visible light will affect both attraction and alignment interaction ranges and intensity. This is the main parameter for the environment that can be controlled.

We have added two paragraphs addressing these points at the very beginning of section 3, which indeed provides clearer motivations for studying the phase diagram of the model (in addition to the obvious theoretical interest). In addition, we have added a short mention of these motivations in the discussion (around line 648).

#3.2 *The paper is tedious to read. More and more parameters, more and more phase diagrams, and more and more intuitions and discussions of this or that effect whose relative importance (by whichever scale) is not really mentioned. I think that this way of presenting the results does not do the work justice. One possible solution is to organize the results and parameters by their effects on relevant behaviors such as the ability to escape from a predator as discussed in the introduction.*

A: We did our best to present as clearly as possible our simulation results: (1) first the different possible states when the intensity of the alignment and attraction interactions change, (2) then more specifically the influence of the interaction range and the fluctuations in individual behavior on these collective states and (3) in the end the impact of group size on collective states. This rigorous, progressive and logical presentation of the results makes it possible to grasp in a simpler way the impact of the various parameters of perception and behavior on collective states. And as shown in Escobedo *et al.* (Ref.44), these different parameters (mostly intensity and range of the alignment and attraction interactions) are essential to understand the different behaviors observed in different species. We think that it is the simplest and the most logical way to present the results as underlined by the two other referees.

#3.3 *It would be nice to mark the region that corresponds to the fitted parameters relevant to H. rhodostomus on all or some of the phase space plots where.*

A: Done.

#3.4 *Specific events that trigger a change in the collective state are shown in the SI movies and shortly mentioned in the discussion but not quantitative analysis of which events trigger a change and which don't – how stochastic this is etc…. this is provided in the results. Results of this sort were previously presented by the Couzin group (https://doi.org/10.1371/) and by the Gov group (https://doi.org/10.1371/). In the discussions the authors suggest that H. rhodostomus may change its collective state by varying some internal parameters – is there any evidence for this? Again, this kind of control can make the paper a more engaging read.*

A: See our answer to point #3.1, and in particular, the 2 new paragraphs added at the beginning/introduction of section 3.

#3.5 *The discussion attributes some of the findings in this paper to the fact that the fish exhibit discrete rather than continuous update (line 574). However, I did not find substantiation for this in the results. Rather, it could be that other characteristics of the model (such as the identity of the most influential fish as the most different one) account for the results. In short, this claim is very interesting and must be substantiated.*

A: First, compared to a continuous model, the discrete model is characterized by an extra characteristic time (the duration of the gliding period after a kick; τ~0.5s), or equivalently, an extra characteristic length (the distance traveled during the gliding period; l~7cm). In addition, the discrete nature of the model results in the fact that for very large intensity of the attraction and/or alignment interaction, the heading angle changes between kicks become essentially random, resulting in the fact that the school cannot remain bounded. This apparently paradoxical lack of cohesion for, say, large attractive interaction would not be observed in a continuous model (the group would get denser and denser as the attractive interaction increases). Note that in a bounded circular tank (characterized by yet another length: its radius), the fact that *H. rhodostomus* (1 or 2 fish) swim most of the time near the wall of the tank has been shown to mainly result from the burst-and-coast nature of the motion for this species (a similar phenomenon arises for bacteria displaying run-and-tumble motion).

In the discussion of these effects, we now explicitly mention the role played by the discrete nature of the model (line 360 and 400).

#3.6 *There are several small prediction verifications mostly hidden in the SI and mostly mentioned in the discussion. I think that analyzing a model to this degree of detail but not testing a single one of its predictions makes the paper somewhat lopsided and not as strong as it could have been. Predictions concerning the species of study but varying group size or water turbidity (as mentioned in the discussion) are certainly possible. The journal policies state that "reliability and significance of biological discovery through computation should be validated and enriched by experimental studies. Inclusion of experimental validation is not required for publication, but should be referenced where possible" So, I would simply urge the authors to highlight any experimental tests/validations to any non-trivial predictions of their model.*

A: As already mentioned, this model was derived by extracting the different interactions from the analysis of actual trajectories of rummy-nose tetra. Since it is anticipated that a change of their environment could modify the main interactions parameters (intensity and range of the

attraction/alignment; see point 3.1 above) of the fish, it is definitely of interest to explore the different collective behaviors emerging from varying these parameters. More generally, since we anticipate that this kind of model (also derived for zebra fish but with different parameters) could have a wide applicability to other fish species displaying a burst-and-coast swimming mode, it is certainly crucial to obtain a better knowledge and understanding of the different possible collective phases emerging in the model by varying the interaction parameters (for instance, the weaker collective behavior generally observed in zebra fish compared to rummy-nose tetra can be directly related to the measured shorter range of interactions). This said, we have added the references mentioned in connection to point #3.1.

ADDITIONAL TEXT AND FIGURES:

We have also added a detailed description of the different kind of transition that there is between the schooling region and the dispersion and swarming regions when γ_{Att} varies across the schooling region for a constant value of γ_{Ali}. The text added to the manuscript is as follows, and there are also two additional figures, S4 Fig and S5 Fig, representing the polarization and the number of groups respectively, corresponding to each value of γ_{Att}:

> *"We performed a series of very long simulations of our model for values of γ_{Att} and γ_{Ali} across the region of high polarization in Fig. 3B, that is, for γ_{Att} going from 0.026 to 0.06 and for a fixed value of γ_{Ali} = 0.2 (20 times longer simulations, until t = 20.000s, each point is the average of 100 runs). S4 Fig shows that the polarization is maximal and remains at its highest value P about 0.85 for a small interval of the attraction strength γ_{Att} in [0.034, 0.04]. Outside this interval, high polarization is lost, and this occurs in two different ways, depending on if γ_{Att} is smaller or higher than the values contained in this interval. For smaller values of γ_{Att}, the polarization decreases because the attraction strength is so small that the group splits in subgroups that move away from each other. Even if fish may be still aligned inside each subgroup, they are not aligned with fish from other subgroups, and consequently the polarization of the total group decreases. For values of γ_{Att} higher than 0.04, the attraction strength is sufficiently high to maintain the group cohesive but in turn makes the fish to rotate excessively towards their neighbor, thus unbalancing the alignment between fish and consequently the polarization of the group. This effect is stronger the higher γ_{Att} is. For larger values of γ_{Att}, above 0.06, the polarization has disappeared and the group is in a swarming state.*
>
> *The transition from high to low polarization as γ_{Att} decreases depends on the duration of the simulations. Each line in S4 Fig represents more than 5 hours of real swimming of the whole fish group, a time during which environmental conditions are expected to vary considerably, at least in light and temperature. Fig. 3 (and the rest of simulations in the paper), each point is the result of simulations of 16.6 minutes of swimming time, a reasonable time duration during which changes in environmental conditions are considered as not affecting fish behavior."*

Following a suggestion made by Reviewer #1 we added figures S6 and S7 showing respectively, the probability distribution function (PDF) of the number of different most influential neighbors (DMIN) at a given time, and in the three important collective phases (swarming; schooling; milling), and the PDF of the number of different nearest neighbors.

FIGURES:

We summarize below the main changes that have been brought to the figures:

Figure 1: The arrow of angle \phi_i^n was inversed in Figure 1B, as pointed out by Reviewer #1.

Figure 6: Labels of vertical axes were misleading, saying "PDF" when in fact the value in the axis corresponds to the value of the magnitude under consideration, "Polarization" or "Milling". We have also adapted the caption of the figure, which now reads:

Polarization (red) and milling (blue) probability distribution functions and mean values (solid lines) along one vertical and two horizontal cuts of the phase diagram for the case k = 1. (AB) Vertical cut at γ_{Att} = 0.0325, (CD) Horizontal cut at γ_{Ali} = 0.2, and (EF) Horizontal cut at γ_{Ali} = 0.05. Each vertical line is the probability distribution function (PDF) of the corresponding value of the

parameter shown in the x-axis, for the parameter of each figure. Solid lines are the corresponding mean value of each PDF. Interaction ranges are l_{Att} = l_{Ali} = 0.28 m and noise intensity is γ_R = 0.2.

Figures 4, 7, and 8: We have corrected the range of values of polarization (red region), from [0.5, 0.85] to [0.4, 0.75], so that they coincide with the one used in Fig. 4. This consistency among figures allows to better compare different cases. We have also added the mention to the cyan region in the caption of Figs. 7 and 8, which was missing, as well as the explicit values of P and M that define each region.

Figures 7, 8, and Figure S1: We have added a yellow dot corresponding to the experimental values of the attraction and alignment strength we measured in *H. rhodostomus*. We have also added, in each caption, the explicit mention to "yellow dots".

In addition to the above-mentioned changes we have also added a new supplementary figure (Figure S4 in the revised version of our manuscript) to illustrate the different kinds of transition between phases observed in Figure 4. Let's start from a value of γ_{Att} well inside the region of high polarization (in red). In the transition from schooling to dispersion as γ_{Att} decreases, the mean value of the polarization decreases because all values of P(t) decreases in time which is a consequence of the fact that the group progressively loses its members with time. In the transition from schooling to swarming as γ_{Att} grows, P(t) is constant in time but smaller as γ_{Att} grows because fish are less and less aligned due to the attraction strength that forces them to point directly towards their most influential neighbor.

Caption of Figure S4: **Time evolution of the mean polarization along extremely long runs for a growing attraction intensity across the schooling phase.** Each line is the average of 100 runs of 20.000 s of duration for different values of γ_{Att} in [0.026, 0.06], *i.e.*, across the schooling region shown in Fig. 4, for γ_{Ali} = 0.2. Line color denote phase region to which the point (γ_{Att} , γ_{Ali}) belongs. Time instants are plotted only each 100 times. The value γ_{Att} = 0.034 seems to be the threshold of the attraction strength below which the curve of the polarization decreases more or less rapidly to the value of no polarization (≈0.1), and above which the curve seems to remain constant in time, at a smaller height at larger values of γ_{Att}.

Caption of Figure S5: **Time evolution of the mean number of 3-groups along extremely long runs for a growing attraction strength γ_{Att} across the schooling phase.**
Each line is the mean number of 3-groups averaged over 100 runs of 20.000 s of duration, for different values of $\gamma_{Att} \in$ [0.026, 0.06], i.e., across the schooling region shown in Fig.4, for γ_{Ali} = 0.2. Line color denotes the phase region in which the point (γ_{Att} , γ_{Ali}) is located. The division of the N fish into 3-groups is done recursively: start with a fish i and put in its group its three nearest neighbors, then the three nearest neighbors of each nearest neighbor of i, and so on. When all the new neighbors of an iteration are already in the group, this 3-group is completed. Take another fish j which is not in a previous 3-group and repeat the process. Once all individuals belong to a 3-group, stop the process and count the number of 3-groups.

Caption of Figure S6: **Probability distribution functions (PDF; solid lines) and corresponding mean values (dashed vertical lines) of the number of different most influential neighbors at a given time in the three collective states, for the case k = 1.**
(A) Schooling state: γ_{Att} = 0.035, γ_{Ali} = 0.2; (B) Milling state: γ_{Att} = 0.04, γ_{Ali} = 0.05; (C) Swarming state: γ_{Att} = 0.07, γ_{Ali} = 0.1. PDFs and mean values are derived from 30 simulation runs, each of 1000 s duration, and sampled at each second in the time interval [200 s, 1000 s] (30 × 800 = 24000 data sampled per graph, leading to an uncertainty for the corresponding mean of order 0.1%). For all cases, the group size is N = 100, the intensity of random fluctuation is γ_R = 0.2, and the social

interaction ranges are l_{Att} = l_{Ali} = 0.28m. We note that there are significantly more different most influential neighbors in the milling state (mean close to 46) than in the 2 other states (mean around 39).

Caption of Figure S7: **Probability distribution functions (PDF; solid lines) and corresponding mean values (dashed vertical lines) of the number of different nearest neighbors at a given time in the three collective states, for the case k = 1.**
(A) Schooling state: γ_{Att} = 0.035, γ_{Ali} = 0.2; (B) Milling state: γ_{Att} = 0.04, γ_{Ali} = 0.05; (C) Swarming state: γ_{Att} = 0.07, γ_{Ali} = 0.1. PDFs and mean values are derived from 30 simulation runs, each of 1000 s duration, and sampled at each second in the time interval [200 s, 1000 s] (24000 data sampled per graph; uncertainty for the corresponding mean less than 0.1%). For all cases, the group size is N = 100, the intensity of random fluctuation is γ_R = 0.2, and the social interaction ranges are l_{Att} = l_{Ali} = 0.28m. We find that the mean number of different nearest neighbors is close to 71 in the three collective states, and is much larger than the corresponding number of different most influential neighbors.
