# Supplementary material for: The impact of individual perceptual and cognitive factors on collective states in a data-driven fish school model
Source: PLoS Comput Biol. 2022 Mar 2;18(3):e1009437. doi: 10.1371/journal.pcbi.1009437 (PMC8932591; doi:10.1371/journal.pcbi.1009437)
Supplement: Attachment — Submitted filename: 0Rebuttal_Letter_Wang_et_al_R2.pdf [file pcbi.1009437.s017.pdf]

Dear Editor,

Please find attached the revised version of our manuscript *"The impact of individual perceptual and cognitive factors on collective states in a data-driven fish school model"* by W. Wang, R. Escobedo, S. Sanchez, C. Sire, Z. Han, and G. Theraulaz.

We acknowledge the fact that Referees #1 and #3 are satisfied with our modifications and with our reply to their comments and address below some additional comments of Referee #2. We now hope that you will consider this article to be suitable for publication.

Sincerely,

The authors

As a general comment, we think that the reviewer did not fully grasp our clearly stated main motivation in this work. Our goal is not merely to find a model in order to just produce some non-trivial collective states (like schooling or milling). Indeed, this can be achieved by many phenomenological models often based on very different (and sometimes, incompatible…) hypotheses, which are not necessarily validated experimentally. Our goal here is to study the possible emergence of such collective phases and the corresponding phase diagrams in a specific realistic data-driven model which was built by reconstructing the interactions from dozens of hours of single fish and pair of fish trajectories [44] and the way individual fish combine pairwise interactions in small groups [60].

In quantum mechanics or animal behavior, "successful" mathematical models are supposed to reproduce (or describe) empirical data and should allow to formulate predictions that can be used to test the validity of the model: the scientific method allows us to only falsify a theory, but not to validate it. So, at a given time, we have at most a best candidate model. My question was about the uniqueness of the model. In my opinion, it is not evident that the data cannot be described using a reduced set of interactions and/or with a different type of formulation. I just suggested the authors to comment on this point.

Our present work is theoretical, so we guess that when the reviewer mentions "data", he/she is referring to our work [44], where the model was built from experiment. Of course, there is not "one" model, and any model is characterized by the extent of the phenomena (see below) it aims at reproducing and its resulting level of complexity.

Although the point here should not be to discuss the work [44], its originality was to introduce a method of **reconstructing the interactions** (described in full detail in [45]) and then directly **plug these resulting interactions into a model** which was ultimately validated successfully in [44] and in [60], using a set of fine observables: probability distribution functions (not just the mean or moments) of the distance of the fish to the wall, of their angle with respect to the wall, of the distance between fish, of the heading angle between fish, of their viewing angles, of the heading angle variation between kicks, polarization, cohesion, …

3. In their answer the authors comment that the model is based on one of their previous publications and that "social interactions functions of fish have been specifically extracted from experimental data [...] don't leave room for manipulation without the risk of losing fidelity of what

is observed in the reality". What are the authors pretending here? Are they suggesting that their model is independent of the choice of observables, dynamic variables, mathematical tools, or types of equation (e.g. standard ODEs, etc.)? Are they suggesting that there exist a unique description of "the reality"? Or do they claim that their model is "the reality"? And by the way, what is "the reality"?

We acknowledge that this sentence in our reply 2.2 was certainly misleading. What we meant again is that our goal was not to just find a model reproducing non-trivial collective states, something that many very different phenomenological models can do, but to study the emergence of such collective states in our data-driven model. Hence, we naturally **keep the very same form of these interactions as found in [44]** and only modulate their intensity and range in the present work, in order to study its collective states and phase diagrams.

To make this point clear we have extended the second paragraph of the introduction (line 53) and added the reference [22] (Barberis L, Peruani F 2016) that we indeed forgot to add in our resubmission.

The level of detail to be incorporated in a model is also a choice. For instance, the burst-and-coast swimming mode can be relevant depending on the question/observable to be tackled, as much as is friction is relevant to understand how a pendulum stops swinging, but we learn a great deal by neglecting it and focusing on short time scales (what is the reality here?). I believe that it is evident that idealized models have proven to be very useful. So, what is the question that the authors try to address that requires the explicit incorporation of the burst-and-coast swimming mode. And is it correct to think that models with explicit burst-and-coast are so fundamentally different from other models when it comes to group polarization and/or cohesion that it is not possible to compare them with previously existing models with no burst-and-coast?

In our previous work [44], we showed that including the burst-and-coast nature of the swimming mode of *H. rhodostomus* was **absolutely essential** to explain and reproduce the fact that one fish or a pair of fish would most often swim close to the wall, a phenomenon also observed in bacteria displaying run-and-tumble motion (see our previous reply to points 3.5 and 3.6 for more details). We have added a sentence to mention this at the end of the second paragraph of section 2.1.1.

Now, in the present work, our goal was to study the possible collective states emerging from this data-driven model. Again, we agree with the reviewer that several phenomenological models (often based on very different ingredients) are able to display schooling or milling states. But that is simply not the point: we are interested in exploring the arising of such collective states in a specific realistic **model which was initially not built to produce these states, but only to capture the behavior of one fish or a pair of fish in a small tank**.

Finally, as pointed out in the discussion section (lines 666-675) the simulation results show that a "burst-and-coast" swimming mode fundamentally changes the dynamics of collective states when the strength of social interactions (attraction and alignment) increases. Indeed, in a model with "burst-and-coast", stronger social interactions do not always enhance group cohesion and coordination while in models with continuous updating rules, cohesion and polarization both increase with the intensity of social interactions [31, 62]. This is a major difference between these models.
